# Deletion of the RNaseIII Enzyme Dicer in Thyroid Follicular Cells Causes Hypothyroidism with Signs of Neoplastic Alterations

**DOI:** 10.1371/journal.pone.0029929

**Published:** 2012-01-05

**Authors:** Wendy Rodriguez, Ling Jin, Véronique Janssens, Christophe Pierreux, Anne-Christine Hick, Eneko Urizar, Sabine Costagliola

**Affiliations:** 1 Institut de Recherche Interdisciplinaire en Biologie Humaine et Moléculaire (I.R.I.B.H.M.), Faculté de Médecine, Université Libre de Bruxelles (ULB), Brussels, Belgium; 2 Christian de Duve Institute (UCL), Brussels, Belgium; French National Center for Scientific Research - Institut de biologie moléculaire et cellulaire, France

## Abstract

Micro-RNAs (miRNAs) are small non-coding RNAs that regulate gene expression, mainly at mRNA post-transcriptional level. Functional maturation of most miRNAs requires processing of the primary transcript by Dicer, an RNaseIII-type enzyme. To date, the importance of miRNA function for normal organogenesis has been demonstrated in several mouse models of tissue-specific Dicer inactivation. However, the role of miRNAs in thyroid development has not yet been addressed. For the present study, we generated mouse models in which Dicer expression has been inactivated at two different stages of thyroid development in thyroid follicular cells. Regardless of the time of Dicer invalidation, the early stages of thyroid organogenesis, preceding folliculogenesis, were unaffected by the loss of small RNAs, with a bilobate gland in place. Nevertheless, Dicer mutant mice were severely hypothyroid and died soon after weaning unless they were substituted with T4. A conspicuous follicular disorganization was observed in Dicer mutant thyroids together with a strong down regulation of Nis expression. With increasing age, the thyroid tissue showed characteristics of neoplastic alterations as suggested by a marked proliferation of follicular cells and an ongoing de-differentiation in the center of the thyroid gland, with a loss of Pax8, FoxE1, Nis and Tpo expression. Together, our data show that loss of miRNA maturation due to Dicer inactivation severely disturbs functional thyroid differentiation. This suggests that miRNAs are mandatory to fine-tune the expression of thyroid specific genes and to maintain thyroid tissue homeostasis.

## Introduction

Congenital hypothyroidism (CH) is one of the most common neonatal metabolic disorder (1∶4000) [1]–[3], characterized by a deficiency in the production of thyroid hormones. If untreated early in life, CH can lead to mental retardation and abnormal growth. CH has been associated with loss of function mutations in thyroid specific genes such as *Sodium Iodide Symporter (NIS)* [*4*] *, Thyroglobulin (TG)* [*5*] *, Thyroperoxidase (TPO)* [*6*], H_2_O_2_ generating system, *DUOX2* [7], and DUOXA2 [8], *Pendrin (PDS)* [9] or *DEHAL1* [10], that result in a reduction of the thyroid hormone production. But the majority of CH cases are due to defects in thyroid organogenesis (ca 85%), with a gland absent (agenesis), abnormally located (ectopy) or severely reduced in size [hypoplasia, as observed for mutations in the *Thyrotropin receptor* gene (*TSHR*) [11] [12], [13]. Unfortunately, the underlying molecular mechanisms are poorly understood.

In most species, thyroid organogenesis can be oversimplified into two phases comprising (a) thyroid precursor cells specification, budding and migration of the thyroid primordium, and (b) functional differentiation of thyroid follicular cells [14]. During mouse embryogenesis, thyroid precursor cells can be identified as a subpopulation of cells at the ventral endoderm of the pharyngeal floor by concurrent expression of four transcription factors (TITF1, PAX8, FOXE1, HHEX) [2]. Genetic invalidation of these transcription factors in mouse showed that they are not required for thyroid specification but that loss of any of them results in thyroid dysgenesis. However, mutations found in *TITF1, PAX8* (See [15] for review), and *FOXE1* [16], together with mutations found in *TSHR* [11] [17] can only explain a low percentage of CH cases with thyroid development abnormalities [2] [18]. This suggests that thyroid developmental transitions are orchestrated by a network of appropriate molecular events which remain elusive [19] [20] [21].

In mammals, growing evidence demonstrates that small non-coding RNAs, and in particular microRNAs (miRNAS), play a substantial role in development [22] [23], cell maintenance and disease [24]–[27]. miRNAs are 22-nucleotides long non-coding RNAs, that regulate gene expression in a variety of tissues more commonly by binding to the 3′UTR of target mRNAs, thereby triggering mRNAs degradation or translational repression [reviewed in [28]. Functional maturation of miRNAs requires processing of the primary transcript by Dicer, an RNaseIII-type enzyme [29]. Thus, in the absence of Dicer, miRNA maturation of most miRNAs is blocked [23]. In turn, inactivation of Dicer provides an efficient approach to examine the function of small RNAs, including miRNA and small interfering RNAs (siRNAs), in specific biological processes. To date, the importance of small RNAs for normal organogenesis (e.g., pancreas, brain, lung, heart, ovary) has been demonstrated in several mouse models of tissue-specific Dicer inactivation [30]–[38]. In the present study, we took a global approach to study the role of small RNAs in thyroid organogenesis by invalidating Dicer in thyroid follicular cells.

We have examined the effect of conditional *Dicer* knockout (cKO) in *Pax8*-Cre and *Tg*-Cre transgenic mice, which enabled deletion of floxed *Dicer* alleles in thyroid follicular cells at E8.5 and E14.5 respectively [2], [39]. In both models, mutant mice die soon after weaning, due to severe hypothyroidism. Dicer inactivation caused thyroid hypoplasia in addition to tissue disorganization and a marked down-regulation of Nis expression. When lethality was delayed in T4 substituted animals, an ongoing de-differentiation of thyroid tissue was observed. Our data show that loss of small RNA maturation due to Dicer inactivation in the thyroid severely disturbs late stages of thyroid organogenesis and progressively results in a cancer-like phenotype. Taken together, our results suggest that small RNAs, likely miRNAs, play an essential role in thyroid homeostasis and raise the possibility that small RNAs may be involved in some human thyroid neoplastic alterations.

## Results

### Thyroid-specific Dicer deletion during early thyroid development; Pax8(Cre/+); Dicer^flox/flox^ transgenic line

**Figure 1:**
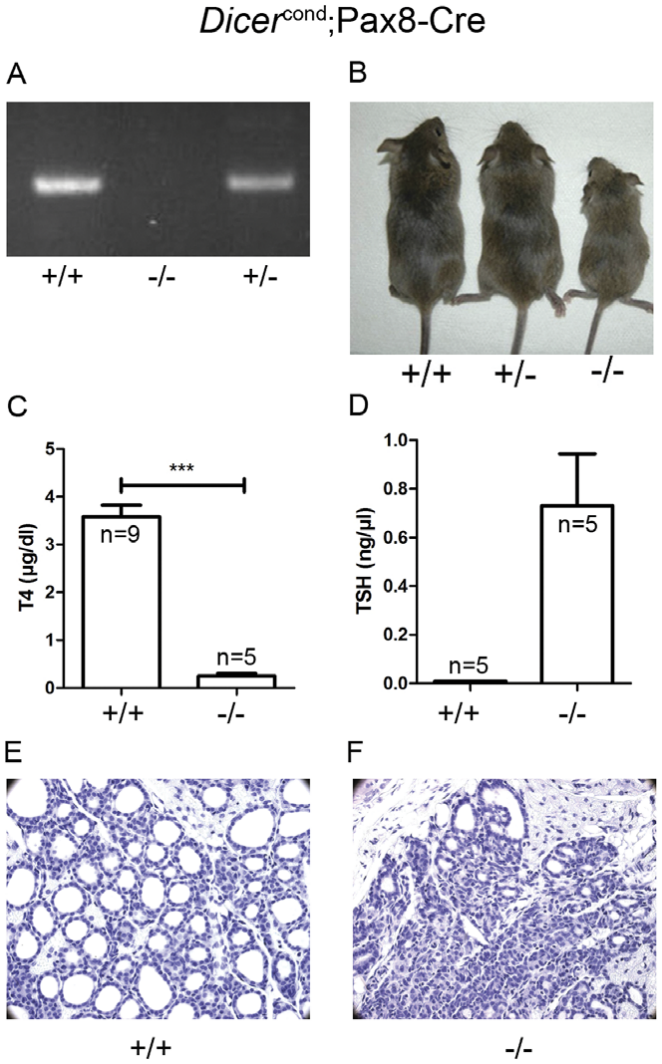
Early conditional Dicer inactivation in the developing thyroid causes hypothyroidism. **A**: To verify that Dicer is inactivated in the thyroid, a PCR was performed in genomic DNA isolated from thyroid from 4 weeks old mice. The different genotypes correspond to: wt (+/+) *Dicer^flox/flox^*, *Pax8(Cre/+)*; *Dicer^flox/flox^* (−/−) and *Pax8(Cre/+)*; *Dicer^flox/+^* (+/−). *Dicer* excision upon *cre* recombination resulted in an absence of a visible fragment that was observed in homozygous mice, whereas there is a single 1,300 bp fragment in the wt and the heterozygous mice. **B**: Growth delay in *Pax8(Cre/+)*; *Dicer^flox/flox^* (−/−) age matched animals. **C**: Low concentration of total blood T4 in *Pax8(Cre/+)*; *Dicer^flox/flox^* mice (p = 0,001, evaluated by a t test). **D**: Elevated TSH in *Pax8(Cre/+)*; *Dicer^flox/flox^* mice. **E, F**: Loss of follicular structure in thyroid sections of *Pax8(Cre/+)*; *Dicer^flox/flox^* (−/−) 3 weeks old mice.

To evaluate the involvement of miRNAs in thyroid development and differentiation, we inactivated Dicer specifically in thyroid follicular cells. In the *Pax8(Cre/+); Dicer^flox/flox^*, Cre is expressed under the control of the *Pax8* promoter therefore based on the thyroid *Pax8* expression pattern (onset on E8.5), we expected Cre-mediated Dicer inactivation early during thyroid development. PCR on genomic DNA obtained from thyroid cells confirmed Pax8-Cre mediated recombination of Dicer resulting in a null Dicer allele, through deletion of exon 24 encoding most of the second RNaseIII domain of the protein (Figure 1A). In addition, Pax8-cDicer mutant mice displayed higher thyroid transcript levels of Dicer^Δexon 24^ than wild type (+/+) or heterozygous (−/+) mice (Figure S1A), showing that, as expected, Dicer mRNA is expressed in mouse thyroid and that *Pax8* driven Cre was efficient to excise the floxed exon 24 of Dicer in thyroid follicular cells. The expression of 10 mature miRNAs (let7i, mir-21, mir-26a, mir-29a, mir-29b, mir-29c, mir-30d, mir-125b, mir-135b, mir-143) was analyzed by TaqMan qPCR from total RNA extracted from thyroids of 3-weeks Pax8-cDicer mutant mice and control mice (n = 2). As expected, Dicer knock-down in thyroid follicular cells reduced the abundance of mature miRNAs. (**, p<0,01;*** p<0,001 unpaired t test) (Figure S1B).

### Profound hypothyroidism, with damaged thyroid architecture in Pax8-cDicer mutant mice

Mice with all possible genotypes were recovered at Mendelian frequency at birth, suggesting that *Dicer* deletion did not cause embryonic lethality. Nevertheless, at three weeks of age, Pax8-cDicer (−/−) mutant mice showed growth retardation (Figure 1B), displayed profound hypothyroidism with low total T4 (0,248±0,126 µg/dl representing Mean±S.D.) and increased TSH (0,731±0,478 ng/µl) plasma levels in comparison to Dicer flox/flox mice (T4 = 3,581±0,729 µg/dl and TSH = 0,01±0 ng/µl). Figure 1C–D). The majority of Pax8-cDicer (−/−) mutant mice did not survive beyond weaning time. The size of the thyroid gland was markedly smaller and pale as compared with control mice and was often characterized by thyroid lobes asymmetrical in size (data not shown). Histological examination of three weeks old mice revealed a profoundly damaged architecture of the thyroid tissue, with a prominent loss of the follicular structure (Figure 1E–F), but to a lesser extent at the periphery of the gland. Follicular cell polarization was analyzed in three weeks old Pax8-cDicer (−/−) mice (Figure S2). In +/+ thyroid sections, follicles showed E-cadherin and ZO-1 staining consistent with mature adhesive and tight junctions, respectively. A similar pattern was observed in Pax8-cDicer thyroid sections, with E-cadherin and ZO-1 staining in cell-cell contact zones. However, a marked difference in the tissue organization of Pax8-cDicer mutant mice was observed with smaller follicles in diameter with a small lumen.

### Expression of thyroid specific genes in 5 days old Pax8(Cre/+); Dicer^flox/flox^ mice

**Figure 2:**
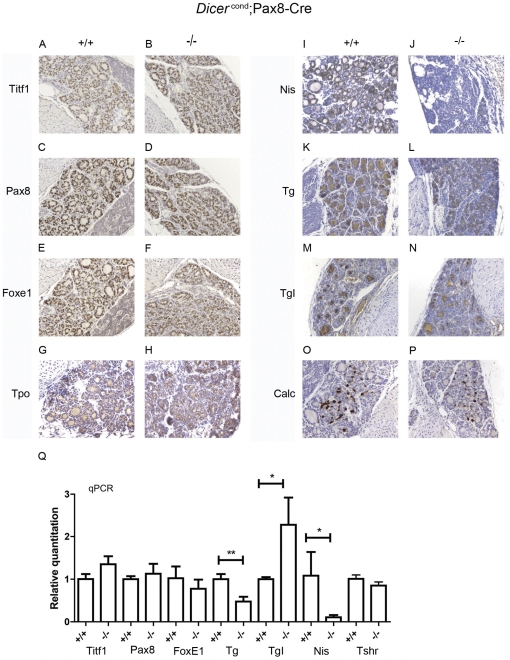
Thyroid specific expression profile in 5 days old animals. Immunohistochemical analysis (see Material and Methods for details) performed in paraffin sections of 5 days old *Dicer^flox/flox^* (+/+) (**A, C, E, G, I, K, M, O**) and *Pax8(Cre/+)*; *Dicer^flox/flox^* (−/−) (**B, D, F, H, J, L, N, P**) mice showed no significant differences in the expression pattern of thyroid specific genes at the protein level, with the exception of NIS (**I, J**) that seemed downregulated in the −/− mice (**J**) when compared to the +/+ mice (**I**). qPCRs showed that most of thyroid protein related transcripts were not downregulated, with the exception of Nis and Tg (**Q**). Statistical significance was evaluated by unpaired t test (*p<0,05; **p<0,005).

Immunohistochemistry studies were performed on thyroid paraffin sections and qRT-PCR on extracted thyroid RNA of 5 days old *Pax8(Cre/+); Dicer^flox/flox^* mice. Absence of functional Dicer in the thyroid did not significantly affect the expression of Titf1, Pax8, FoxE1, three transcription factors involved in thyroid development (Figure 2A–F for protein expression and Figure 2Q for mRNA level). Among thyroid specific markers, only Nis exhibited a strongly reduced expression both at protein (not detectable; Figure 2I–J) and mRNA levels (Figure 2Q). Tg expression is slightly reduced (Figure 2 K–J and -Figure Q), whereas expression of Tpo (Figure 2 G–H), iodinated Tg (Figure 2M–N), Tshr (Figure 2Q) are maintained. C-cells number, identified by Calcitonin expression, was not affected (Figure 2O,P).

### Thyroid specific gene downregulation, increased proliferation and general de-differentiation in T4 substituted one month old Pax8(Cre/+); Dicer^flox/flox^ mice

**Figure 3:**
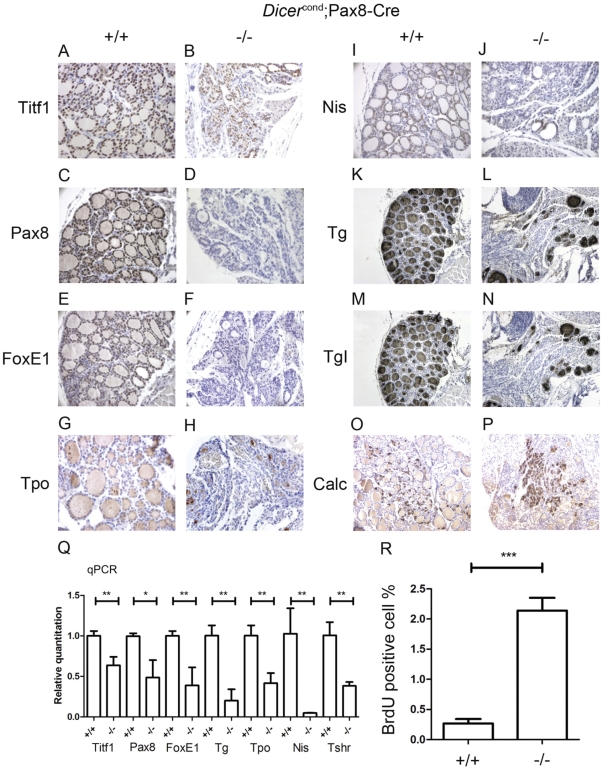
Thyroid dedifferentiation occurs after one month in early Dicer inactivated animals. Immunohistochemical analysis performed as explained for Figure 2 in paraffin sections of 4 weeks old *Dicer^flox/flox^* (+/+) (**A, C, E, G, I, K, M, O**) and *Pax8(Cre/+)*; *Dicer^flox/flox^* (−/−) (**B, D, F, H, J, L, N, P**) mice showed a significant reduction in all the studied thyroid related genes at the protein level, with the exception of Ttf1 (**A, B**) and calcitonin (C-cell marker) (**O,P**). A qPCR (see Material and Methods) was performed on total thyroid RNA extracts from one month old mice. Results are expressed as fold of the control (+/+) condition. The RNA expression for most of thyroid genes was significantly downregulated in *Pax8(Cre/+)*; *Dicer^flox/flox^* mice (−/−) (**Q**) (p>0,005, evaluate by umpaired t test). The proliferation rate, evaluated by a BrdU staining was strongly increased in 4 weeks old *Pax8(Cre/+)*; *Dicer^flox/flox^* (−/−) compared to *Dicer^flox/flox^* (+/+) (p<0,0001, t test) littermates (**R**).

Most of *Pax8(Cre/+); Dicer^flox/flox^* mice usually die around weaning time due to hypothyroidism. Surprisingly and in contrast to what is observed in *Pax8* null or *hyt/hyt* hypothyroid mice [2], [39], where T4 substituted mice survive to adulthood, death was only delayed for a couple of weeks in Pax8-cDicer mice under thyroid hormone replacement therapy. In rare mice surviving one or two extra weeks, the thyroid progressively de-differentiated and immunohistochemistry revealed marked changes for most thyroid specific markers (Figure 3). Whereas Titf1 protein expression appeared marginally downregulated (Figure 3A–B), an almost complete absence of Pax8 (Figure 3C–D) and FoxE1 (Figure 3E–F) protein staining was observed. Tpo (Figure 3G–H) and Nis (Figure 3I–J) proteins were barely detectable in most of the thyrocytes. Tg and TgI expression was altered, with a staining mostly restricted to the thyrocytes of remaining follicular structures, located at the periphery of the thyroid sections (Figure 3K–N). In contrast, calcitonin staining of C-cells was unaltered (Figure 3O–P). Western blot analysis of TiTF1, Pax8, FoxE1 and Tg confirmed the immunohistochemical observations (Figure S3). TiTF1, Pax8, FoxE1, Tg, Tpo and Nis mRNA levels measured by qPCR confirmed the immunohistological and western blot observations (Figure 3Q). In addition, Tshr mRNA level appeared downregulated as well (Figure 3Q). BrdU incorporation identified a marked increase of the proliferation rate in *Pax8(Cre/+); Dicer^flox/flox^* mice (2,14±0,665%) in comparison to *Pax8(Cre/+); Dicer^flox/+^* mice (0,268±0,162%) (Figure 3R). Nevertheless, the number of follicular cells was not significantly different between thyroids of Pax8-cDicer mutant mice (619±52,07) and wt mice (801±31,07).

### Thyroid-specific Dicer deletion in later stages of thyroid development; Tg(Cre/+); Dicer^flox/flox^ transgenic line

**Figure 4:**
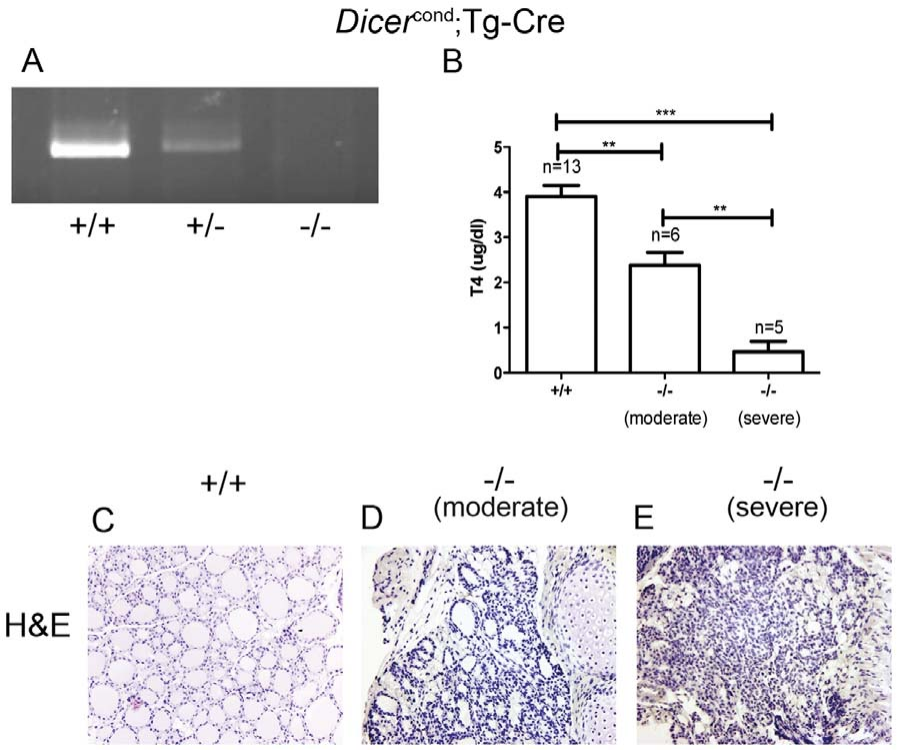
Conditional invalidation of Dicer late during thyroid development uncovers surprisingly severe hypothyroidism. **A**: As for *Pax8(Cre/+)*; *Dicer^flox/flox^* (−/−) mice, a PCR was performed in genomic DNA issued from thyroid from 4 weeks old mice. The different genotypes correspond to: wt (+/+) *Dicer^flox/flox^*, *Tg(Cre/+)*; *Dicer^flox/flox^* (−/−) and *Tg(Cre/+)*; *Dicer^flox/+^* (+/−). *Dicer* excision upon *cre* recombination resulted in an absence of visible fragment that was observed in homozygous mice, whereas there was a single 1,300 bp fragment in the wt and the heterozygous mice. **B**: Total blood T4 was lower in *Tg(Cre/+)*; *Dicer^flox/flox^* 3 weeks old mice (***, p<0.001; **, p<0.01, unpaired t test). Hematoxylin staining on paraffin sections on 3 weeks old *Dicer^flox^* ^/*flox*^ (+/+) (**C**) and *Tg(Cre/+)*; *Dicer^flox/flox^* (−/−) (**D, E**) mice showed two different phenotypes for the −/− animals. In half of the Dicer KO litters, the follicular structure was moderately altered, but still present (**D**), and in the other half the follicular structure was lost (**E**).

To study the role of miRNAs in later stages of thyroid development, an additional thyroid follicular cell-specific conditional cKO mouse line, *Tg(Cre/+); Dicer^flox/flox^*, was generated (Tg-cDicer, −/−). Those mice express Cre recombinase under the control of the mouse *Tg* promoter (onset E14.5). *Tg(Cre/+); Dicer^flox/+^* mice were crossed with *Dicer^flox/flox^* to generate progeny with inactivated Dicer during later stages of thyroid development. As observed in *Pax8(Cre/+); Dicer^flox/flox^ mice*, PCR analysis of genomic DNA extracted from thyroid revealed an efficient excision of the floxed *Dicer* exon by Cre recombinase (Figure 4A) and *Tg(Cre/+); Dicer^flox/flox^* genotype did not disrupt Mendelian distribution of the offspring. When examined at three weeks of age, and in contrast to *Pax8(Cre/+); Dicer^flox/flox^* mice, *Tg(Cre/+); Dicer^flox/flox^* mice could be grouped into two different categories according to their phenotype (Figure 4B–E). A first viable group presented a “moderate phenotype” with regard to overall health (T4 plasma levels were moderately reduced, 2,393±0,495 µg/dl, in comparison to *Dicer^flox/flox^* mice, 3,805±0,715 µg/dl) Figure 4B) and the degree of thyroid alterations (Figure 4C–D). In these mice with a moderate phenotype, Nis is the only markedly downregulated gene being only detectable in the few follicles with appreciable lumen (data not shown). The second group displayed a severe phenotype comparable to that observed in age-matched *Pax8(Cre/+); Dicer^flox/flox^* mice, with hypothyroidism associated with low T4 plasma levels (0,47±0,386 µg/dl, Figure 4B), disruption of thyroid architecture (Figure 4E) and post-weaning lethality. Unexpectedly and similarly to what we observed with Pax8-cDicer mice, Tg-cDicer substituted with T4 rarely survived to adulthood.

### Expression of thyroid specific genes in 5 days old Tg(Cre/+); Dicer^flox/flox^ mice

Immunohistochemistry studies were performed on thyroid paraffin sections and qRT-PCR on extracted thyroid RNA (from two independent litters) of 5 days old *Tg(Cre/+); Dicer^flox/flox^* mice. At this developmental stage, mice invalidated for Dicer presenting with moderate phenotype cannot be distinguished from mice with severe phenotype.

**Figure 5:**
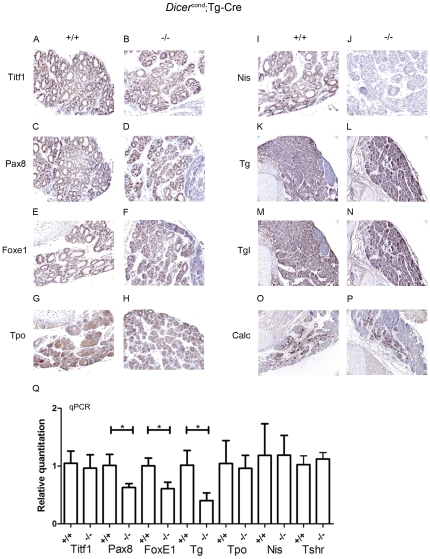
Most of thyroid specific transcripts were not affected in 5 days old Tg(Cre/+);Dicer^flox/flox^ mice. Immunohistochemical analysis performed as explained for Figure 2 in paraffin sections of 5 days old *Dicer^flox/flox^* (+/+) (**A, C, E, G, I, K, M, O**) and *Tg(Cre/+)*; *Dicer^flox/flox^* (−/−) (**B, D, F, H, J, L, N, P**) mice showed no significant reduction in all the studied thyroid related genes at the protein level, with the exception of Nis (**I,J**) that was not detectable. qPCR (**Q**) revealed that most of the thyroid related transcripts were not affected by Dicer inactivation. Nevertheless, we observed a slight decrease of Pax8, FoxE1 and Tg mRNA level in Tg(Cre/+); Dicer^flox/flox^ (−/−) in comparison to Dicer^flox/flox^ (+/+) mice.

Dicer invalidation in the thyroid at later stages markedly affected the expression of Nis protein (not detectable; Figure 5I–J) whereas mRNA levels for this marker remained unchanged (Fig. 5Q). We cannot exclude the possibility that such a discrepancy between Nis protein and mRNA levels could be a consequence of the heterogeneous phenotype exhibited by this animal model (see above). 5 days after birth, conditional deletion of functional Dicer in *Tg(Cre/+); Dicer^flox/flox^* mice did not significantly affect the protein level of other thyroid markers (Figure 5 A–H and K–N). Whereas no difference in Titf1, Tpo, Nis and TSHr mRNA levels could be detected (Figure 5Q), Pax8, FoxE1, Tg mRNA levels were reduced (Figure 5Q) by 40% (Pax8 and FoxE1) to 60% (Tg) by comparison with wild type animals. No apparent differences in expression were observed for other thyroid markers at the protein level (Figure 5), despite a significant reduction in Pax8, FoxE1 and Tg mRNA levels (Figure 5Q). C-cell number, identified by Calcitonin immunoreactivity, remained unaltered (Figure 5O–P).

### Expression of thyroid specific genes in one month old Tg(Cre/+); Dicer^flox/flox^ mice

Consistent with *Pax8(Cre/+); Dicer^flox/flox^* mice, an ongoing de-differention of the thyroid tissue was observed with *Tg(Cre/+); Dicer^flox/flox^* mice. Immunohistochemistry studies were performed on thyroid paraffin sections and qRT-PCR on extracted thyroid RNA of one month old *Tg(Cre/+); Dicer^flox/flox^* mice presenting with a “severe” phenotype (see above).

**Figure 6:**
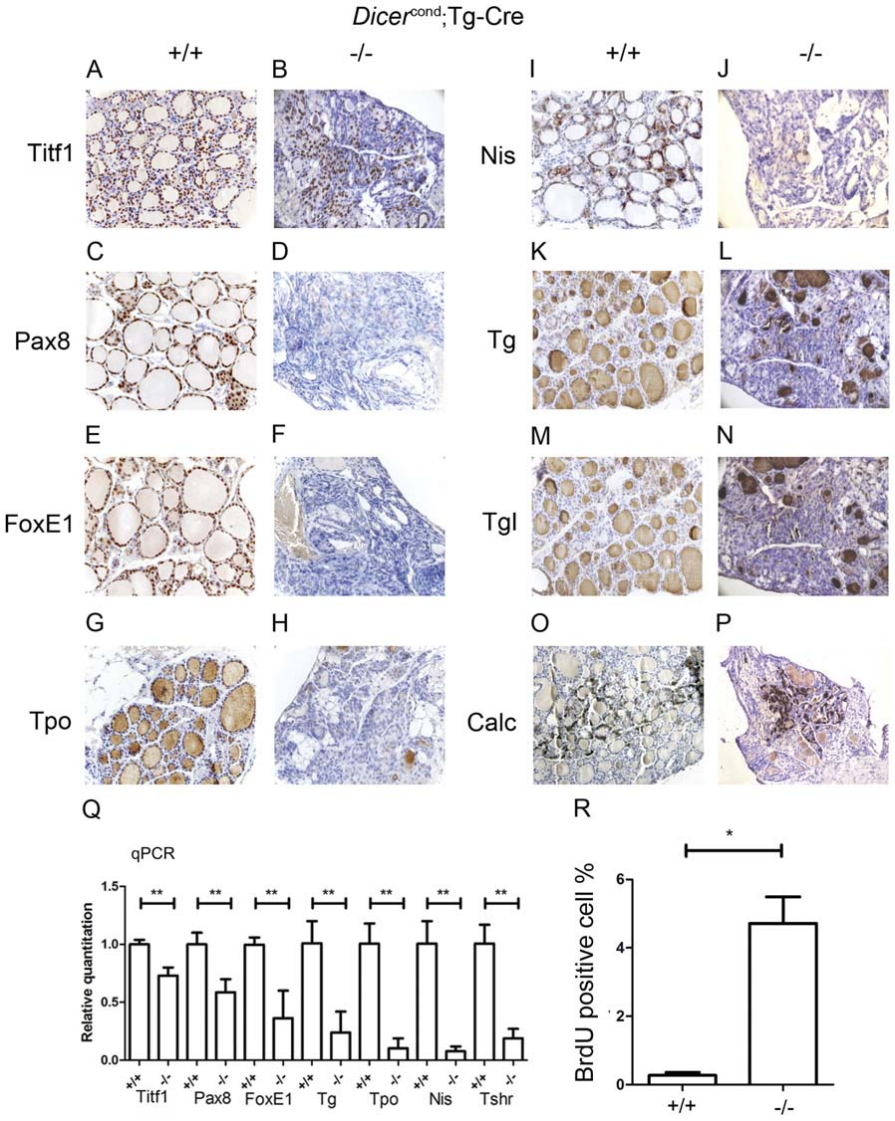
Dedifferentiation occurs also when Dicer is inactivated late during thyroid development. Immunohistochemical analysis performed as explained for Figure 3 in paraffin sections of 4 weeks old *Dicer^flox/flox^* (+/+) (**A, C, E, G, I, K, M, O**) and *Tg(Cre/+)*; *Dicer^flox/flox^* (−/−) (**B, D, F, H, J, L, N, P**) mice showed a significant reduction in all the studied thyroid related genes at the protein level, with the exception of Titf1 (**A, B**) and calcitonin (Calc) (**O, P**) that were not downregulated. qPCR was performed in total RNA extracts from thyroids of one month old mice and results are expressed relative to the control condition (+/+) (**Q**). *FoxE1*, *Nis* and *Tpo* expression was significantly downregulated in *Tg(Cre/+)*; *Dicer^flox/flox^* mice (−/−) (p<0,001, evaluated by umpaired t test). The proliferation rate, evaluated by a BrdU staining as explained for Figure 4, is strongly increased in 4 weeks old *Tg(Cre/+)*; *Dicer^flox/flox^* (−/−) compared to *Dicer^flox/flox^* (+/+) (p<0,05 umpaired t test) littermates (**R**).

A lack of detectable Pax8 and FoxE1 protein expression (Figure 6C–F), and sporadic Tpo and Nis protein expression (Figure 6G–J) were observed in the thyroid gland with increasing age. Interestingly, Titf1 protein expression appeared marginally affected (Figure 5A–B). A decrease of Tg and TgI protein expression was observed as well, especially in the central zone of the gland (Figure 6K–N). Calcitonin expression was not affected (Figure 6O–P). Modulations of mRNA expression levels were consistent with immunohistochemical protein detection (Figure 6Q), except for Pax8, for which a complete absence of protein immunoreactivity was accompanied by a 40% reduction of the mRNA level. Tshr mRNA level was also severely reduced (73%) (Figure 6Q). A marked increase of proliferation rates was observed in *Tg(Cre/+); Dicer^flox/flox^* mice (4,77±0,77%) in comparison to *Tg(Cre/+); Dicer^flox/+^* mice (0,27±0,09%) (Figure 6R) and as already observed in Pax8-cDicer mutant mice, the number of follicular cells was not significantly different between thyroids of Tg-cDicer mutant mice (585±39) and wt mice (801±31).

## Discussion

Small RNAs regulate several developmental processes [22] [27]. The aim of this work was to dissect the role of Dicer and small RNAs-mediated gene regulation in thyroid development and differentiation. We inactivated Dicer in thyroid follicular cells, early during thyroid development or at later stages, at the beginning of folliculogenesis [2]. One month after birth, the first major defect observed in the thyroid of both Dicer mutant models was a marked follicular disorganization, which impaired efficient thyroid hormone production and caused profound hypothyroidism. Indeed, organization of thyroid follicular cells into follicles is mandatory for thyroid hormones production. Those follicles are the morphofunctional units of thyroid gland, formed by a monolayer of polarized cells facing at the apical surface an internal compartment, the follicle lumen, containing colloid. After iodide uptake mediated by Nis protein at the basal membrane, iodination and thyroid hormone synthesis occur at the apical plasma membrane thanks to Tg, Tpo and Duox/Duoxa proteins.

Interestingly, even if a preliminary analysis of the architecture of the thyroid tissue was interpreted as a lack of follicular structures, staining of polarity markers showed the presence of follicles but without appreciable luminal compartment. At this stage, most thyroid markers were downregulated at the mRNA level in *Dicer* invalidated animals. In addition, in both models, Pax8, FoxE1, Tpo and Nis proteins were almost undetectable. Interestingly, TSHr mRNA levels were also markedly reduced in thyroid of one-month-old animals, suggesting that the increase of the proliferation rate observed was likely not a consequence of a Tshr mediated cAMP signalling hyperstimulation.

How to interpret the presence of follicles without appreciable follicular lumen? It has been reported that bidirectional ionic (Na^+^/Cl^−^) transport in thyroid follicles influences the follicular size by determining fluid accumulation in the follicular lumen [40]. In the *Dicer* mutant thyroid, a disturbed fluid transport could be responsible for the observed follicular disorders. Alteration of fluid dynamics would limit Tg secretion and thus prevent primordial follicle opening [41]. In agreement with such notion, in conditional inactivated Dicer thyroid Tg appeared partly trapped in the thyrocyte cytoplasm. However, rare Nis positive mature follicles with enlarged lumina are also present in the thyroid of *Dicer* mutants. These follicles, often observed at the periphery of the gland may have escaped *Dicer* recombination or may have been rescued by a different mechanism. Despite a strong Cre-mediated alkaline phosphatase staining reported in the thyroid gland of *Pax8(Cre/+)*; *Z/AP* embryos or *Tg(Cre/+)*; *Z/AP* embryos, [42] [43], that some thyroid areas simply did not undergo complete *Dicer* recombination cannot be ruled out (Figure S1).

On the other hand, reported evidence for non cell-autonomous small RNA action could explain this heterogeneous phenotype [38], [44]. In agreement with such a notion, the few formed follicles are in close proximity with the so called “thyroid capsule” which contains neural and vascular structures [45]. In addition, small RNAs produced by surrounding tissue like parathyroid glands, often embedded in the thyroid parenchyma, could also locally neutralize and compensate for the effects of *Dicer* invalidation in a subset of thyrocytes. Taken together, these observations demonstrate that Dicer invalidation in the thyroid shortly after specification (onset E8.5) does not impair early development of the gland but does affect final follicular maturation. Indeed, *Dicer* mutants are characterized by thyroid hypoplasia accompanied by abnormal tissue morphology. A low number of follicles with appreciable lumen could account for the thyroid hypoplasia.

Interestingly, late *Dicer* invalidation phenocopies the thyroid defect observed in mice where *Dicer* was invalidated early during development. Indeed, in the mouse model where Cre-expression was driven by a *Tg* promoter, severe hypothyroidism was observed and was associated with early post-weaning lethality. The persistence of lethality in T4 complemented mice is puzzling. Cre recombinase expression was not reported at extra thyroidal sites in Tg-Cre mice ([43] and personal communication). Therefore an eventual toxic effect of Cre expression or Dicer invalidation in another organ can be ruled out. Moreover, Pax8-cDicer −/− and Tg-cDicer −/− mice die at the same age and in the same condition, leading us to imagine a common cause so far unidentified. Histopathological and biochemical analysis of body fluids and tissue other than thyroid should help to a better understanding of the lethal phenotype.

In severely hypothyroid *Tg(Cre/+); Dicer^flox/flox^* mice (see below), the thyroid presented profound tissue disorganization, with a low number of follicles presenting a large luminal compartment, reminiscent of the thyroid in the animals where Dicer was evaluated earlier during thyroid development. This demonstrated that *Dicer* invalidation early or late during thyroid development could trigger highly comparable phenotypes.

In this model of *Dicer* inactivation at a later stage, close to the time when folliculogenesis is initiated, the extent of the observed defects was variable among the mutant mice. Indeed, some *Tg-cDicer* −/− animals exhibited a moderate phenotype with respect to the overall health and the degree of the thyroid alterations. Despite a down-regulation of Nis expression, T4 plasma levels moderately decreased and most individuals survived after weaning in the absence of a T4 supplement. This relatively less severe phenotype may be solely a timing issue. In this conditional *Dicer* loss of function mouse model, Cre transcription occurs around E15 [43], at the time when the thyroid primordium is undergoing thyrocyte differentiation and follicular organization. It has been reported that a delay between the onset of Cre expression and the actual biological effect can vary by few hours [46]. In our model we also need to consider the time required for mRNAs degradation and/or translational repression [47]. Whereas this would have no impact among Pax8-cDicer mice, because *Dicer* is invalidated before thyroid differentiation, such a gap could trigger marked discrepancies between Tg-cDicer phenotypes, because Dicer invalidation occurs concomitantly with the final maturation of the follicular cells. At this stage, the fine-tuning of the miRNA gene network is probably mandatory for the regulation and/or selection of the molecular events that will dictate final identity of the thyroid cell.

Despite an altered follicular structure and a marked Nis downregulation, most thyroid specific markers are present in follicular cells five days after birth, irrespective of the time when *Dicer* was invalidated. Interestingly, in benign cold follicular adenomas, a decrease of *NIS* mRNA levels was reported, whereas the expression of *TG*, *TSHR* and *TPO* was maintained [48]. Indeed, the low expression of Nis present in the *Dicer* invalidated animals, may represent an early abnormality in the transformation pathway of the thyroid cell, and not be a consequence of a neoplastic progression. Could we nevertheless interpret the phenotype observed in *Dicer* mutant thyroid as a beginning of thyroid de-differentiation? To answer this question using our approach, thyroid phenotypes were analyzed at later stages (one month after birth) in *Dicer* mutant animals supplemented with T4. In both models, an increased damage of the follicular tissue architecture was observed, together with a loss of appreciable expression of Nis and additional thyroid specific markers (Pax8, FoxE1, Tpo, Tg, Tshr) at mRNA and/or protein level, suggesting an ongoing process of thyroid de-differentiation. In both animal models, follicular cell proliferation was also markedly increased. Interestingly, in mutant mice, Tg and TgI expression appeared to be increased in rare follicles positive for those two markers. While we cannot exclude some staining artifacts, we could speculate for a direct consequence of a lost miRNA. Nevertheless, while Dicer invalidation revealed global requirements for miRNAs in the thyroid, this approach is unable to pinpoint roles for direct and specific mRNA-miRNAs interactions that could be crucial for thyroid development and function.

In every cell type, loss of differentiated functions is associated with carcinogenesis. During human thyroid tumorigenesis, thyroid specific genes display a specific pattern in their alteration, with a primary loss of Nis expression subsequently followed by that of Tpo, Tg and Tshr [49]. Such a scenario is nicely recapitulated in our animal models with a prominent loss of Nis expression, consistent with a marked downregulation of Tshr measured at the mRNA level, followed by a decrease of Tpo. At the protein level, Tg expression appears more resistant to the de-differentiation process and the protein was undetectable only in the areas with no appreciable follicular structures. Titf1, Pax8 and FoxE1 transcription factor modification is known to preclude or modify a normal expression of thyroid specific genes such as *Nis*, *Tpo* and *Tg* at the transcriptional level, thus conferring to those transcription factors a key role in the oncogenic process. Indeed, several studies reported a reduced *TiTF1*, *PAX8* or *FOXE1* expression in human thyroid tumors, especially in the poorly differentiated carcinomas [50], [51]. In the *Dicer* mutant thyroid, a marked loss of Pax8 and FoxE1 expression was observed. Pax8 is a master gene for the regulation of the thyroid-differentiated phenotype [52] and both Pax8 and FoxE1 are essential for the hormonal control of *Nis* and/or *Tpo* gene transcription [2]. Indeed, their concomitant down-regulation could explain the loss of these two-target genes. In contrast, in Dicer mutant thyroid tissue, Titf1 protein is still well detected. There is evidence for TiTF1 and Pax8 cooperation in the transcriptional activation of the *Tg* promoter in thyroid cells [53]. In our Pax8-cDicer mutants, where Pax8 expression appears mandatory to maintain Tpo and Nis expression, TiTF1 alone seems sufficient to trigger Tg expression. In addition to the loss of expression of several thyroid specific genes, invalidation of *Dicer* has a marked effect on follicular cell proliferation, despite a low level of Tshr mRNA. miRNAs are known to regulate the expression of genes involved in the control of proliferation [54] and there is evidence that suggests a critical role of miRNAs down-regulation in thyroid carcinogenesis [55].

Taken together, our data demonstrate an essential role of small RNAs in the thyroid to damp fluctuations in target mRNAs that could compromise correct thyroid follicular cell differentiation and lead to cancer initiation or/and progression.

## Materials and Methods

### Ethics Statement

The institutional ethics committee (CEBEA-Commission d'Ethique du Bien Etre Animal) of the Université Libre de Bruxelles, approved the animal experiments used in this study, permit number 342N.

### Generation of Dicer1 thyroid mutant mice

Mice expressing a *Dicer* conditional allele (*Dicer^flox^* ^/flox^) [30] were crossed with mice expressing *Pax8(Cre/+)* transgene [42] to obtain mice expressing *Pax8(Cre/+)*; *Dicer^flox^* ^/+^. Those mice were crossed with *Dicer^flox^* ^/flox^ in order to obtain *Dicer* conditional knockout (cKO) mice [*Pax8(Cre/+)*; *Dicer^flox^* ^/flox^]. In parallel, another Dicer cKO line was created by using mice expressing cre recombinase under the control of the mouse thyroglobulin (Tg) promoter following a similar strategy [43]. Offspring were genotyped by using PCR (for Pax8(Cre/+) product sizes: 389 wt and 673 mutant; for Tg(Cre/+) product sizes: no amplification for wt and 493 bp for mutant and for Dicerflox/flox product sizes: 351 wt and 420 mutant; primers are available upon request). Using genomic DNA from thyroid, the deleted allele was genotyped by using primers DicerF1 and DicerDel (5′-CCTGAGCAAGGCAAGTCATTC-3′). The wt PCR product resulted in a 1,300-bp segment whereas no amplification was observed when the exon 24 of dicer was deleted.

### T4 and TSH dosage

Total T4 was measured by RIA with the *Coat-A-Count Canine T4* kit (Siemens Medical, USA) according to the manufacturer procedure. TSH was quantified by ELISA with a Rat TSH kit (Shibayagi, Japan).

### Histology, Immunohistochemistry and Immunofluorescence

Fixed (4% PAF) paraffin tissue sections were rehydrated in an ethanol gradient and subsequently blocked in 1% BSA-1% donkey serum containing PBS for one hour at room temperature (RT). Primary antibody solution diluted appropriately in blocking buffer [1∶2000 rabbit anti-Titf1, 1∶700 rabbit anti-Foxe1, 1∶3000 rabbit anti-Pax8 (Biopat, PA 0100, PA 0200, PA 0300, Italy), 1∶1000 rabbit anti-Nis (N. Carrasco, USA), 1∶2000 rabbit anti-Tpo (F. Miot, Belgium), 1∶2000 mouse anti-TgI (C.Ris-Stalpers, The Netherlands), 1∶3000 rabbit anti-Tg (Dako, A0251, Denmark) and 1∶8000 rabbit anti-Calci (Dako, A0576, Denmark)] was incubated overnight (ON)). A solution with a biotinylated secondary antibody (Jackson ImmunoResearch, United Kingdom) diluted in blocking buffer was incubated for one hour at RT after extensive washes of the primary antibody. After washing the secondary antibody, slices were incubated 30 mins with a HRP complex and revealed. Hematoxylin staining was done immediately after.

### Study of proliferation

0.05 mg/g mouse weight 5-Bromo-2′-deoxyuridine (BrdU) (Sigma, USA) was injected 3 hours prior to sacrifice. Tissue processing was done exactly as described above and proliferation index was determined by estimating the ratio of BrdU positive and total cells. For each mouse 10 thyroid slices have been quantified for BrdU incorporation. In addition, all follicular cells were counted on each slice. Statistical significance was evaluated by a unpaired t test.

### Quantitative PCR on thyroid extracts

Total RNA was extracted from thyroid mice using *mirVana miRNA Isolation Kit* (Ambion, AM1560) and RT-PCR generated cDNA was amplified. Quantitative PCR was performed using Brilliant II Fast SYBR qPCR Master Mix (Agilent Technologies) on a real-time PCR system (Mx3005P; Agilent Technologies). All primers were designed using Lasergene 7.2 software (DNAStar, Inc.) and are available upon request. TATA-binding protein (*TBP*) was used as endogenous control. Relative expression levels of these genes were determined by the delta-delta threshold cycle (ΔΔC_T_) method and statistical significance was evaluated by unpaired t test.

For analysis of mature miRNA expression, cDNA was synthesized from 10 ng of total RNA (isolated as described above) using the TaqMan MicroRNA Reverse Transcription Kit with miRNA-specific RT primer from the TaqMan MicroRNA Assay Mix. miRNA levels were measured using the miRNA-specific TaqMan probe provided in the MicroRNA Assays and the TaqMan Gene Expression Maser Mix (Applied Biosystems). Experiments were performed in triplicate on a real-time PCR system (Mx3005P; Agilent Technologies). miRNA levels were normalized to *U6* snRNA (Applied Biosystems) and fold change was determined by the comparative threshold method (ΔΔC_T_). Statistical significance was evaluated by unpaired t test.

## Supporting Information

Figure S1 **Dicer excision upon cre recombination and expression levels of mature miRNAs. A)** PCR was performed in cDNA from thyroids of 3 weeks old mice with primers that amplify exon 23 to 25. The different genotypes correspond to: wt (+/+) *Dicer^flox/flox^*, *Pax8(Cre/+)*; *Dicer^flox/+^* (+/−); *Pax8(Cre/+)*; *Dicer^flox/flox^* (−/−). *Dicer* excision upon *cre* recombination results in a 199 bp fragment that is observed in homozygous mice, whereas there is a single 431 bp fragment in the wt mice and both fragments are amplified in the heterozygous animals. Presence of wt size *Dicer* transcripts were also detected in homozygous Pax8-cDicer (−/−) mutant mice, probably due to contamination of the tissue by C-cells and cells from the parathyroid gland and vasculature, but a partial *Pax8(Cre/+)* mediated recombination cannot be excluded. **B)** TaqMan qPCR to analyze expression levels of mature miRNAs. DICER as induced global miRNA expression deficits in thyroid of Pax8-cDicer (−/−) mutant mice compared to WT mice. Relative expression levels of miRNAs were determined by the delta-delta threshold cycle (ΔΔC_T_) method and normalized to the endogenous *U6* snRNA. Two independent experiments (n = 2) were performed in triplicate. Statistically significant changes in the relative levels of miRNA expression were observed (***, p<0.001;**, p<0.01, unpaired t test).
(TIFF)

Figure S2 **Follicular structure disruption with normal cell polarity in mice with early conditional Dicer1 inactivation.** **A, B**: Lost of Folicular structure in thyroid sections of *Pax8^cre/-^*; *Dicer^flox/flox^* (−/−) 3 weeks old mice. E-cadherin (**C, D**) and ZO-1 (**E, F**) immunofluorescence in paraffin sections of 3 weeks old mice shows intact basolateral and apical polarity are maintained in −/− mice (**D, F**) despite of the disrupted follicular structure.
(TIF)

Figure S3 **Western blot analysis of thyroid markers in 1-month old** ***Pax8^cre/-^*** **;** ***Dicer^flox/flox^*** **(−/−) mice.** Whereas no difference in Titf1protein levels could be detected, Pax8, FoxE1, Tg protein levels were reduced by comparison with wild type animals (+/+). See Material and Methods S1 for detailed western blot protocol.
(TIF)

Materials and Methods S1 **Detailed protocol of western blot.**
(DOC)
