# Supplementary figures and images for: Deletion of the RNaseIII Enzyme Dicer in Thyroid Follicular Cells Causes Hypothyroidism with Signs of Neoplastic Alterations
Source: PLoS One. 2012 Jan 5;7(1):e29929. doi: 10.1371/journal.pone.0029929 (PMC3252359; doi:10.1371/journal.pone.0029929)

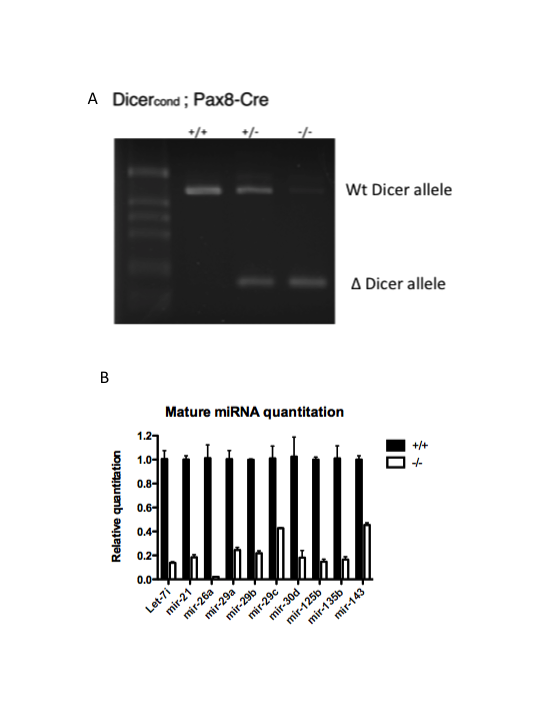

Supplement: Figure S1 — Dicer excision upon cre recombination and expression levels of mature miRNAs. A) PCR was performed in cDNA from thyroids of 3 weeks old mice with primers that amplify exon 23 to 25. The different genotypes correspond to: wt (+/+) Dicerflox/flox, Pax8(Cre/+); Dicerflox/+ (+/−); Pax8(Cre/+); Dicerflox/flox (−/−). Dicer excision upon cre recombination results in a 199 bp fragment that is observed in homozygous mice, whereas there is a single 431 bp fragment in the wt mice and both fragments are amplified in the heterozygous animals. Presence of wt size Dicer transcripts were also detected in homozygous Pax8-cDicer (−/−) mutant mice, probably due to contamination of the tissue by C-cells and cells from the parathyroid gland and vasculature, but a partial Pax8(Cre/+) mediated recombination cannot be excluded. B) TaqMan qPCR to analyze expression levels of mature miRNAs. DICER as induced global miRNA expression deficits in thyroid of Pax8-cDicer (−/−) mutant mice compared to WT mice. Relative expression levels of miRNAs were determined by the delta-delta threshold cycle (ΔΔCT) method and normalized to the endogenous U6 snRNA. Two independent experiments (n = 2) were performed in triplicate. Statistically significant changes in the relative levels of miRNA expression were observed (***, p<0.001;**, p<0.01, unpaired t test). (TIFF) [file pone.0029929.s001.tif]

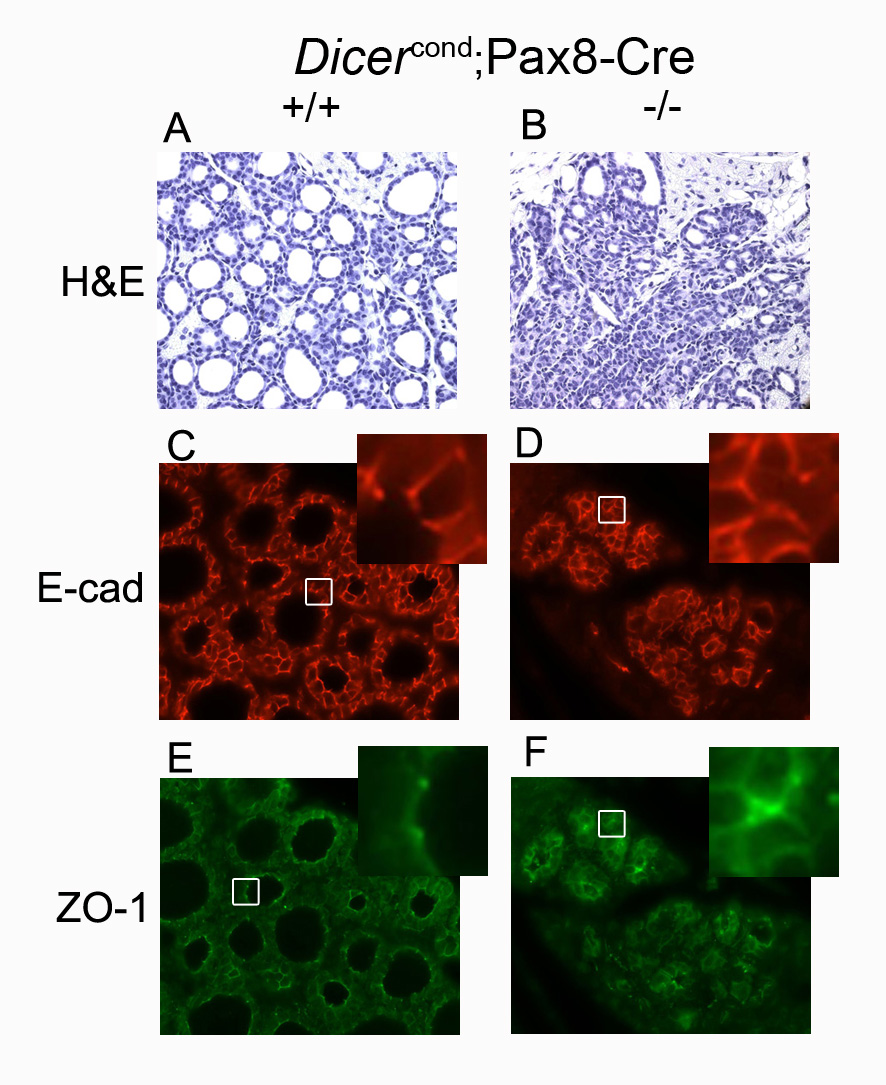

Supplement: Figure S2 — Follicular structure disruption with normal cell polarity in mice with early conditional Dicer1 inactivation. A, B: Lost of Folicular structure in thyroid sections of Pax8cre/-; Dicerflox/flox (−/−) 3 weeks old mice. E-cadherin (C, D) and ZO-1 (E, F) immunofluorescence in paraffin sections of 3 weeks old mice shows intact basolateral and apical polarity are maintained in −/− mice (D, F) despite of the disrupted follicular structure. (TIF) [file pone.0029929.s002.tif]

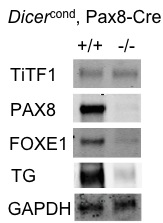

Supplement: Figure S3 — Western blot analysis of thyroid markers in 1-month old Pax8cre/- ; Dicerflox/flox (−/−) mice. Whereas no difference in Titf1protein levels could be detected, Pax8, FoxE1, Tg protein levels were reduced by comparison with wild type animals (+/+). See Material and Methods S1 for detailed western blot protocol. (TIF) [file pone.0029929.s003.tif]
