# Supplementary material for: Deletion of the RNaseIII Enzyme Dicer in Thyroid Follicular Cells Causes Hypothyroidism with Signs of Neoplastic Alterations
Source: PLoS One. 2012 Jan 5;7(1):e29929. doi: 10.1371/journal.pone.0029929 (PMC3252359; doi:10.1371/journal.pone.0029929)
Supplement: Materials and Methods S1 — Detailed protocol of western blot. (DOC) [file pone.0029929.s004.doc]

**Material and Methods S1**

**Western blot analysis**

Thyroids of one month old mice were lysed in 10 mM Tris–HCl pH 7.4, 0.1%SDS0, 0.5% NP-40, 150 mM KCl, 12 mM β-mercaptoethanol, 100 mM NaF, complete protease inhibitor tablet (Roche, Mannheim, Germany), 0.01 mg/ml leupeptin, 0.3 mg/ml pefabloc, 1 mM vanadate. Protein concentration of the clarified lysate was measured by the Bio-Rad Protein Assay using bovine serum albumin (ICN Biomedicals Inc., Aurora, USA) as standard. 10 µg proteins of the homogenates were separated by SDS-PAGE gel and transferred to nitrocellulose membrane (Hybond-ECL, Amersham Biosciences). The proteins were detected with the corresponding primary antibodies. GAPDH was immunodetected with a polyclonal antibody (Cell Signaling Technologies, catalog number 2118) to verify that equal quantities of proteins were loaded on the gel. Fluorescent secondary antibodies (1/10,000; IRDye 800 anti goat IgG from Licor, Lincoln, NE, USA, DyLight 680 anti rabbit IgG or DyLight 800 anti mouse IgG from Thermo Fisher Scientific, Rockford, USA) were used for image acquisition with the Odyssey infrared imaging system (LICOR,Lincoln, NE, USA).
